# Supplementary material for: Effectiveness of online training in improving primary care doctors’ competency in brief tobacco interventions: A cluster-randomized controlled trial of WHO modules in Delta State, Nigeria
Source: PLoS One. 2024 Feb 22;19(2):e0292027. doi: 10.1371/journal.pone.0292027 (PMC10883549; doi:10.1371/journal.pone.0292027)
Supplement: Attachment — Submitted filename: Response to Reviewers.docx [file pone.0292027.s002.docx]

Department of Community and Public Health,

Idaho State University,

921 South 8^th^ Avenue, Stop 8109,

Pocatello, Idaho 83209, United States.

11^th^ January 2024.

Billy Morara Tsima, MD, Msc

Academic Editor

PLoS ONE

Dear Dr. Tsima,

**REBUTTAL LETTER: PONE-D-23-28937**

# I humbly wish to express my gratitude to you and the reviewers for your diligent and insightful contributions toward making our manuscript better.

# We have revised the manuscript to meet PLOS ONE’s style requirements, included new copies of Tables 1 – 4 in the manuscript, and uploaded new copies of Figures 1 and 2. We have also prepared a mark-up copy of the manuscript that highlights the changes made to the original version, as well as an unmarked version of the revised paper without tracked changes. Our data set is uploaded as a Supporting Information file. All corrections have been implemented. However, there are some exceptions and we would like to make clarifications on these.

# Reviewer #1

# *Study setting: Are all doctors involved in the care of smokers*

# The World Health Organisation (WHO) Framework Convention on Tobacco Control (FCTC), other relevant documents, and experts have recommended that brief tobacco interventions be routinely offered in primary care and offered to smokers at every contact with a clinician, and that competency in this intervention is an expected standard of practice for every clinician. Our study population was primary care doctors, so they fall within the category of people who should be involved in tobacco intervention.

# *Selection/eligibility criteria: The authors mentioned that “those who were scheduled to proceed on in-service training…”*

# There appears to be some misunderstanding here with the use of “in-service training”. By this, we meant people who would be proceeding on study leave. So, we excluded them because of their likely unavailability for our intervention and data collection. Our intervention was not an “in-service training” in the sense that was elaborated by the reviewer. We appreciate the learning point and have used more appropriate terminology in that part of the manuscript.

# Thank you!

# Yours sincerely,

Dr. Nnamdi Moeteke

[nnamdimoeteke@gmail.com](mailto:nnamdimoeteke@gmail.com)
